# Detailed molecular characterisation of acute myeloid leukaemia with a normal karyotype using targeted DNA capture

**DOI:** 10.1038/leu.2013.117

**Published:** 2013-05-24

**Authors:** N Conte, I Varela, C Grove, N Manes, K Yusa, T Moreno, A Segonds-Pichon, A Bench, E Gudgin, B Herman, N Bolli, P Ellis, D Haddad, P Costeas, R Rad, M Scott, B Huntly, A Bradley, G S Vassiliou

**Affiliations:** 1 Wellcome Trust Sanger Institute, Wellcome Trust Genome Campus, Hinxton, Cambridge, UK; 2 EMBL-European Bioinformatics Institute, Cambridge, UK; 3 Instituto de Biomedicina y Biotecnología de Cantabria, University of Cantabria, Santander, Spain; 4 Bioinformatic Department, Babraham Institute, Cambridge, UK; 5 Department of Haematology, Cambridge University Hospitals NHS Foundation Trust, Hills Road, Cambridge, UK; 6 Agilent Technologies, IQ Winnersh, Reading, UK; 7 Centre for the Study of Haematological Malignacies, Nicosia, Cyprus

**Keywords:** acute myeloid leukaemia, diagnosis, classification, targeted capture, next generation sequencing, minimal residual disease, MIDAS

## Abstract

Advances in sequencing technologies are giving unprecedented insights into the spectrum of somatic mutations underlying acute myeloid leukaemia with a normal karyotype (AML–NK). It is clear that the prognosis of individual patients is strongly influenced by the combination of mutations in their leukaemia and that many leukaemias are composed of multiple subclones, with differential susceptibilities to treatment. Here, we describe a method, employing targeted capture coupled with next-generation sequencing and tailored bioinformatic analysis, for the simultaneous study of 24 genes recurrently mutated in AML–NK. Mutational analysis was performed using open source software and an in-house script (Mutation Identification and Analysis Software), which identified dominant clone mutations with 100% specificity. In each of seven cases of AML–NK studied, we identified and verified mutations in 2–4 genes in the main leukaemic clone. Additionally, high sequencing depth enabled us to identify putative subclonal mutations and detect leukaemia-specific mutations in DNA from remission marrow. Finally, we used normalised read depths to detect copy number changes and identified and subsequently verified a tandem duplication of exons 2–9 of *MLL* and at least one deletion involving *PTEN*. This methodology reliably detects sequence and copy number mutations, and can thus greatly facilitate the classification, clinical research, diagnosis and management of AML–NK.

## Introduction

Advances in DNA sequencing technologies are revolutionising our understanding of the genetic basis of cancer.^1^ One of the first cancers studied by whole-genome sequencing was acute myeloid leukaemia with a normal karyotype (AML–NK),^2, 3^ a disease whose molecular aetiology was, until recently, poorly understood. As a result, we now know of more than 10 genes mutated in >5% of cases of AML–NK and of several others mutated less often.^4, 5, 6^ Additionally, it has become clear that mutations other than those affecting *FLT3*,^7^ *NPM1*^8^ and *CEBPA*^9^ have a significant impact on prognosis and can help stratify anti-AML therapy for individual patients.^4^ In this light, many are calling for a shift towards a classification system for AML–NK based primarily on mutational profiling.^4^

Currently, many diagnostic laboratories routinely screen for mutations in *NPM1* and *FLT3*, both of which show clustering of somatic mutations in 1–3 exons. However, mutational screening for genes such as *CEBPA* and *TET2*, which do not exhibit mutation clustering, is only employed in specialist laboratories. Furthermore, with the identification of an increasing number of mutant genes in AML, detailed molecular genotyping can no longer be practicably performed using conventional molecular methods such as capillary sequencing or melt curve analyses. Moreover, modern sequencing technologies have demonstrated that many cases of AML are composed of several related subclones, arising through the acquisition of different somatic mutations during clonal evolution from a single-ancestral cell.^6, 10^ These clones are often invisible to conventional diagnostic methods, yet they commonly represent a significant, if not the main, clone at the time of leukaemia relapse.^10^ As relapse is the main vehicle for the poor prognosis of AML, the detection of clones carrying adverse mutations at the time of diagnosis can help identify and stratify high-risk patients.

Given the above, a full molecular diagnostic evaluation of AML requires the identification of all mutations with prognostic or therapeutic significance in the main clone, as well as in subclones when these are present. Here we successfully employ targeted DNA capture with cRNA baits followed by deep sequencing and tailored informatics to simultaneously study 24 genes known to be recurrently mutated in AML–NK and 10 control genes.

## Materials and methods

### Leukaemic DNA samples

DNA samples from total bone marrow cells, excess to diagnosis, were obtained after informed consent within our ethics-approved study (07/MRE05/44) from seven patients with AML–NK. Remission samples were obtained from two of these patients and a relapse sample from one.

### Bait design

We designed a set of Sure Select cRNA biotinylated oligonucleotide baits (Agilent Technologies, Palo Alto, CA, USA) to capture all exons from a set of 24 genes known to be recurrently mutated in AML and 10 control genes, some known to be mutated in solid tumours (Table 1). The custom bait library was designed using eArray software (Agilent Technologies). The exons of the 34 genes were downloaded from Biomart (http://www.ensembl.org/biomart/martview/) and used to create 120 bp baits starting every 24 bp. The masking option used was ‘RepeatMasker' and the software allowed baits to overlap by a maximum of 20 bp with repeat masked regions. The centred design strategy was used, which ensured that the tiling level was maintained and baits were not ‘squeezed' in the specified interval. As a result, the input region could be expanded by 20 bp at each end. The concentration of individual baits was adjusted manually depending on the target nucleotide composition to optimise DNA capture GC-rich regions (*n*=1579) had 2x and orphan regions (*n*=649), defined as those covered by a single bait, 5x more bait molecules per locus than standard regions (*n*=5997). The total target region size was 24 2051 nucleotides and the library design is available under the unique ELID reference: 0324251.

### DNA target selection by ‘pull-down'

DNA fragmentation, library preparation and solution phase hybrid capture were performed according to manufacturer's instructions (Agilent Technologies) and modified from previously published protocols.^11^

### Sequencing and mapping

We sequenced 10 samples on a single multiplexed lane on an Illumina HiSeq 2000 and aligned the resulting reads to the hg19 reference genome with BWA (Burrows–Wheeler Alignment; http://bio-bwa.sourceforge.net/bwa.shtml).^12^

### Coverage and statistical analysis

Coverage histograms and tables describing the coverage distribution for our set of targeted bases were produced using TEQC, ‘Target Enrichment Quality Control' Bioconductor package.^13^ To validate the ability of our assay to identify copy number changes, we used read numbers of two X-linked genes (*HPRT* and *KDM6A*). First, we generated a list of non-redundant ‘amalgamated exons', each representing all overlapping annotated exons. Read count normalisation was done using open-source software and bespoke R scripts: for each sample, read counts per position were calculated using Bedtools 2.12.0 (http://code.google.com/p/bedtools),^14^ then normalised read counts were calculated by averaging the exon-specific read counts and dividing by the total number of mapped reads for that sample. As DNA quality can affect capture efficiency and thus read counts, we first wanted to ensure all 10 samples gave comparable standardised read depths for the majority of target regions. For this, we looked at the average read count for each patient at each gene. Sample P5 was an outlier for 23 of the 34 genes and was removed from copy number calculations. All other samples were outliers for three genes or less (Supplementary Figure S1). To identify copy number variation at individual exons, we calculated the coefficient of variability for each exon for the nine patients. We then used the Tukey boxplot approach to identify the outlier exons (>upper quartile+1.5*IQR, interquartile range). Data from genes with an increased coefficient of variability at more than one exon were examined manually. The mixed-lineage leukaemia (*MLL*) deletion was also detected by analysis with ExomeCopy (http://www.bioconductor.org/packages/2.11/bioc/html/exomeCopy.html).

### Mutation calling

#### Alignment and post-processing

Fastq files were aligned against the human genome (hg19 version) using BWA algorithm (v 0.5.9). Afterwards SAMTOOLS (0.1.18) view, sort, index and fixmate algorithms were used to generate, sort, index and fix co-ordinates of the generated bam files. PICARD (v1.61) java libraries were used to mark PCR duplicates and finally GATK (v. 1.4.20) tools were used to perform local realignment around indels. All these steps were automated using a single in-house written script available upon request.

#### Variant calling

**Figure 1:**
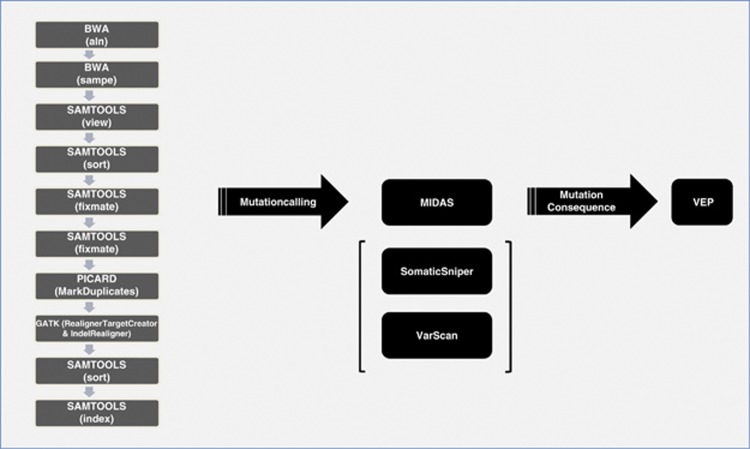
Workflow diagram for data analysis and mutation calling. After initial parsing of sequencing data through a series of open source software tools, mutation calling is performed by our in-house Perl script (MIDAS). Mutational consequences are then determined by Variant Effect Predictor, Ensembl. For the purposes of comparing MIDAS with other callers, SomaticSniper and VarScan were used instead.

SAMTOOLS pileup command was used to generate pileup files from the generated bam files (version 0.1.8) (http://samtools.sourceforge.net/).^15^ A flexible in-house Perl script (MIDAS, Mutation Identification and Analysis Software; available upon request) was created to parse the pileup file and to take into account in each position only those reads with a sequence quality higher than 25 and a mapping quality higher than 15, and consider only those positions that had a coverage of at least 10 both in the tumour and in the control sample (unless otherwise stated, P1CR was used as control for all comparisons). On those positions, and taking into account the high coverage obtained in this experiment, we reported the possible existence of a substitution whenever there was at least 20 independent reads reporting a different base vs the reference genome in the tumour sample and less than 5% of the reads reporting the same variant in the control sample. We also discarded those positions with at least one-third of this evidence reporting a third allele, as we consider that those regions would probably represent difficult sequences for the aligner and would likely produce false positives. We considered variants present in >20% of reads as those representing the main/dominant leukaemic clone. In the case of indels (small insertions and deletions), we considered positive those regions with at least 10 independent reads reporting the same indel in the tumour sample and with less than 5 reads in the control sample, and with at least 10 times more reads reporting the indel in the tumour vs the control sample. Similarly to what we did with substitutions, those regions with an evidence of a second indel higher than 40% of the evidence for the primary indel were discarded. Our workflow is shown in Figure 1. Of note, MIDAS allows adjustment of tolerance thresholds to suit the type of control sample used (for example, they can be increased to facilitate the use of a remission sample as a control, which may harbour residual low-level mutant reads).

#### Comparison with other software/algorithms

The performance of our software was checked using independent variant calling algorithms. In particular, we run SomaticSniper (v. 1.0.2; http://gmt.genome.wustl.edu/somatic-sniper/current/)^16^ on the bam files using the default parameters and VarScan (v2.3, http://varscan.sourceforge.net/)^17^ on the pileup files using both the default mode and a high-sensitivity mode setting a minimum variant frequency of 0.01, a normal purity of 0.95 and a tumour purity of 0.20. In order to be able to compare the results with the calls made by our software, the raw data generated by the other callers were afterwards filtered according to the frequency and ratios criteria specified in the above paragraph.

The predicted protein consequences of variations were derived using Variant Effect Predictor from Ensembl, http://www.ensembl.org/info/docs/variation/vep/index.html.

### Validation of mutations and copy number changes identified by next-generation sequencing

All dominant clone mutations were confirmed using PCR and capillary sequencing. PCR was performed with Platinum Taq Polymearse (Invitrogen Corporation, Carlsbad, CA, USA) for 35 cycles at 56 °C annealing and 72 °C extension for 30 s. To amplify across the breakpoint of the MLL-partial tandem duplication, we used LongAmp 2x Taq mastermix (New England Biolabs, Ipswich, MA, USA) for 35 cycles at 57 °C annealing and 65 °C extension for 3 min. PCR for detection of FLT3-internal tandem duplication was performed as described previously.^18^ Mutant reads were visualised using IGV (Integrative Genomics Viewer; http://www.broadinstitute.org/igv/bam). To verify the two *PTEN* deletions, we used six known single-nucleotide polymorphisms within introns of the *PTEN* gene. We amplified these by PCR, followed by second-round PCR with barcoded Illumina adapter primers and sequencing on a MiSeq sequencer. We used these results to look for evidence of copy number change for one of the two alleles compared with a reference normal (P6 vs ctrl) or a paired remission sample (P2 vs P2CR). All primer sequences are given in Supplementary Table S1.

## Results

**Figure 2:**
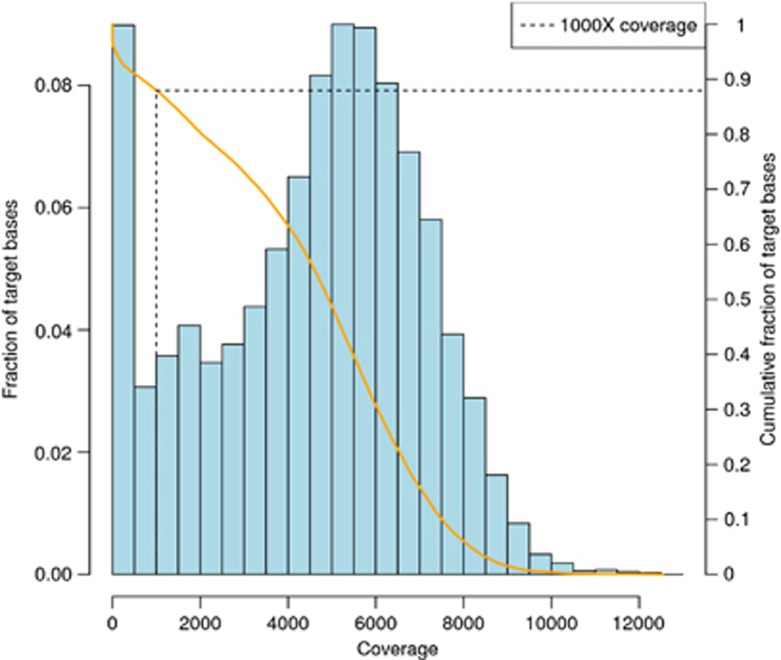
Distribution of the depth of sequencing coverage of the target genes. Representative data from sample P1 showing the fraction of bases covered at incremental depth windows (blue bars and left hand *y* axis) and the cumulative fraction of bases covered at or above the specified coverage (orange line and right hand *y* axis). This shows that ∼88% of bases were covered at by at least 1000x sequencing reads.

Analysis of our sequencing data showed a mean coverage depth of 5136 × per nucleotide position within the target region (Figure 2). The 10 × and 100 × coverage were 96.4% and 94.8%, respectively, for the desired target region (that is, all exons of 34 genes) (Supplementary Table S2), with most of the remaining 3.6–5.2% representing repetitive regions for which baits could not be designed. With regards to substitutions and indels among the seven AMLs studied, our mutation caller, MIDAS, identified 20 exonic and one intronic mutations in the main leukaemic clone (2–4 mutations per AML, Table 2). The same 20 exonic mutations were identified by the VarScan platform and all were successfully validated using Sanger sequencing (Supplementary Figure S2), giving both MIDAS and VarScan 100% specificity for this data set. SomaticSniper, which was designed for the identification of substitutions but not indels, performed slightly less well (Supplementary Table S3). Of the 20 exonic mutations, 11 were single-base non-synonymous substitutions at known sites (9 missense and 2 nonsense) and 9 were small indels (8 associated with premature termination and 1 with a single amino-acid insertion).

In order to determine whether the read depth for our target regions correlated with DNA copy number, we compared standardised read numbers for two X-linked genes, *HPRT* and *KDM6A*, between male and female cases, and contrasted this with the same ratio for the remaining (autosomal) genes. This demonstrated that female cases displayed approximately twice the number of normalised reads of male cases for the two X-linked genes (F:M ratios of 2.0 for *KDM6A* and 1.91 for *HPRT*), signifying that read numbers approximately reflect copy number in the starting DNA. In keeping with this, male and female cases gave similar normalised read numbers (M:F ratios close to 1) for the 32 autosomal genes (Supplementary Figure S3). Samples that deviated from this ratio were later found to harbour copy number variation at the relevant gene locus in one or more samples (for example, *CYP2D6* and *PTEN*). Furthermore, the quantitative nature of the data was evident at the level of individual exons and not just whole genes (for example, Supplementary Figure S5), demonstrating that the data were quantitative even at the level of small independently captured loci.

**Figure 3:**
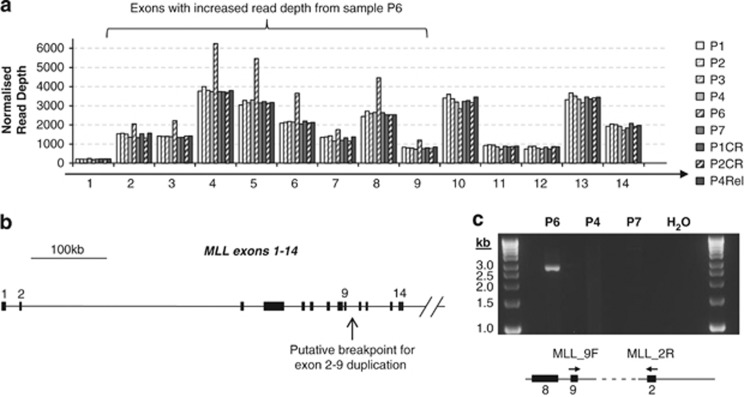
Identification of *MLL* partial tandem duplication (PTD) using sequencing read depth. Normalised per exon sequencing read depths for the first 14 exons of *MLL* show increased depth for exons 2–9 from sample P6 (**a**). This suggested the presence of an exon 2 to exon 9 PTD with a breakpoint in intron 9 (**b**). PCR amplification across the putative breakpoint using an exon 9 forward (MLL_9F) and an exon 2 reverse (MLL_2R) primer confirms the presence of the PTD in this AML sample (**c**).

Given the above, we went on to look for copy number aberrations involving the target exons using the Tukey Box-plot method. The only autosomal gene loci exhibiting a significantly increased coefficient of variability at multiple exons were *CYP2D6*, *MLL* and *PTEN* (Supplementary Figure S4). *CYP2D6* is known to exhibit copy number variation and per exon read numbers were in keeping with one individual (P3) having a lower *CYP2D6* copy number than the others (Supplementary Figure S5b). In the case of *PTEN*, two samples (P2 and P6) had lower read numbers (Supplementary Figure S5c), suggesting that these two cases of AML may harbour deletions involving *PTEN*. To confirm this using our limited material, we looked at differential allelic read counts for six intronic single-nucleotide polymorphisms within the *PTEN* locus, using PCR amplification followed by sequencing on a MiSeq sequencer. Our results confirm copy number change at the *PTEN* locus for P2 by demonstrating a preferential reduction in read counts from one allele of two independent informative single-nucleotide polymorphisms when compared with the matched remission sample (P2CR) (Supplementary Table S4). In the case of P6, only one single-nucleotide polymorphisms was informative and although this was suggestive of copy number loss, we cannot be completely confident this is the case in the absence of a matched normal sample. In the case of *MLL*, one sample (P6) showed an increased number of normalised reads for exons 2–9 only, suggesting the presence of a partial tandem duplication (Figures 3a and b and Supplementary Figure S5d). This was also identified by analysis using the ExomeCopy package^19^ (Supplementary Figure S6). The presence of a partial tandem duplication was confirmed using PCR primers to amplify the region spanning the junction (Figure 2c).

We went on to analyse our data to identify single-nucleotide substitutions uniquely present in putative leukaemic subclones representing as few as 1% of cells. We identified putative subclonal mutations representing 3–20% of reads in four leukaemic samples: (i) a *FLT3* internal tandem duplication in sample P3, which was flagged as a series of indels and substitutions and confirmed by PCR (we went on to test all seven AML samples for *FLT3*-internal tandem duplication and only sample P3 was positive—data not shown), (ii) *NRAS*-G12S and *PTPN11*-Q506P mutations in sample P5. The latter two mutations occurred in 4.4% and 4.1% of reads, respectively, in keeping with possible co-occurrence in the same subclone, (iii) *FLT3*-N676K in sample P4 and (iv) *TP53*-G374fs*8 in sample P4Rel (Supplementary Table S5). Finally, we analysed the two paired diagnosis-remission samples (P1 vs P1CR and P2 vs P2CR) to look for evidence of residual mutant reads in each remission sample. Both remission samples were in morphological complete remission, but sample P1CR was taken after four courses and sample P2CR after one course of chemotherapy. At a level of sensitivity of at least 0.1%, we found no mutant reads in sample P1CR, while sample P2CR gave residual mutant reads representing 1.5–2.4% of total reads for all three mutations identified at diagnosis (Supplementary Table S6).

## Discussion

Advances in sequencing technologies are revolutionising cancer research with somatic mutations underlying most major cancers being avidly identified and characterised in large numbers of cases. Concurrently, clinical and functional studies are defining the diagnostic/prognostic significance of mutations and determining their molecular effects in order to device targeted therapeutic strategies. AML is at the forefront of such progress, and as a result a significant body of information has already been gathered about this leukaemia that can be used to guide clinical practice.

To date, most diagnostic laboratories use allele-specific technologies to identify mutations in genes such as *NPM1* and *FLT3*, which have validated prognostic and therapeutic significance.^7, 20, 21^ Additionally, newer technologies have been shown to reliably identify leukaemia-associated mutations in larger numbers of amplicons using pyrosequencing.^22^ However, the increasing number of clinically relevant genes found mutated in AML make conventional amplicon-based approaches impractical, particularly as many such genes can harbour mutations in multiple different locations and exons.^23, 24^ Additionally, the clear demonstration that many AMLs are composed of multiple subclones that can be differentially susceptible to existing therapies^10^ suggests that accurate therapeutic stratification of patients would benefit from the identification of such clones at first presentation.

We describe a method based on targeted DNA capture with cRNA baits followed by deep sequencing that enables the simultaneous identification of mutations in 24 AML genes, including the 10 most frequently mutated in AML–NK, without recourse to normal constitutional DNA from the same individual. Somatic mutations in the dominant leukaemic clone were identified in all cases studied using sequence alignment/configuration with open source software followed by mutation calling using our in-house mutation caller MIDAS (Figure 2). The same mutations were identified by the mutation caller VarScan^17^ and all mutations so identified were validated using capillary sequencing, demonstrating 100% specificity for both callers for our data set. By contrast, no novel polymorphisms or mutations were identified in 10 control genes known to be mutated in solid tumours or leukaemias other than AML.

The sequencing depth reached in this study also enabled us to identify putative mutations present in subclones at the time of diagnosis. It is already clear that, compared with the main clone, such subclones may be differentially sensitive to chemotherapy and can expand to become the dominant clone at the time of disease relapse,^10^ making their identification at the time of diagnosis important. Nevertheless, at this stage, such subclonal mutations need to be validated using independent methodologies as it remains possible that they represent sequencing or other forms of error.

Additionally, after confirming that our data behaved in a quantitative manner with respect to input DNA copy number in 9 of 10 DNAs studied, we went on to identify copy number variants in leukaemic samples, including an instance of *MLL*-partial tandem duplication and two instances of probable loss of *PTEN*, one of which we were able to validate. In analysing these data it became clear that the lack or copy number information from neighbouring genomic regions made analysis more difficult, and we recommend that future studies of this kind endeavour to capture several features around regions of possible copy number loss to enhance both the power and the reliability of analyses. Finally, we were able to demonstrate evidence of minimal residual disease in a bone marrow DNA sample in morphological complete remission by mining reads from the specific mutations in the remission sample. Quantification of minimal residual disease after induction chemotherapy may have prognostic implications in a heterogeneous disease such as AML–NK and could be employed in interventional studies to determine its significance.

We describe a molecular diagnostic method that enables extensive molecular characterisation of AML–NK at diagnosis and can facilitate clinical management of patients as well as clinical research into this disease. The approach is powerful, reliable and can be introduced into routine clinical practice in order to enhance our ability to identify patients at high risk of relapse as well as those that would benefit from molecularly directed therapies and can also be adapted for minimal residual disease monitoring. The sequencing methodology is modular and target regions can be increased to include any newly discovered gene mutations without significant changes to laboratory protocols and with only marginal increases in costs. Additionally, we provide a clear analytical workflow employing MIDAS, a novel mutation calling algorithm available on request, which correctly identified 20/20 exonic mutations present in >20% of reads. The blueprint presented here can be used to study other haematological or solid tumours, or groups of tumours with overlapping mutational spectra.

## Figures and Tables

**Table 1:** Genes analysed by targeted capture

*Gene ID*	*Chromosome*	*Position (Mb)*
*AML genes*		
*NRAS*	1	115.2
*DNMT3A*	2	25.5
*SF3B1*	2	198.3
*IDH1*	2	209.1
*KIT*	4	55.5
*TET2*	4	106.1
*CSF1R*	5	149.4
*NPM1*	5	170.8
*EZH2*	7	148.5
*JAK2*	9	5.0
*PTEN*	10	89.6
*WT1*	11	32.4
*MLL*	11	118.3
*CBL*	11	119.1
*KRAS*	12	25.4
*PTPN11*	12	112.9
*FLT3*	13	28.6
*IDH2*	15	90.6
*TP53*	17	7.6
*NF1*	17	29.4
*CEBPA*	19	33.8
*ASXL1*	20	30.9
*RUNX1*	21	36.2
*KDM6A*	X	44.7
		
*Control genes*		
*UGT1A1*	2	234.7
*PIK3CA*	3	178.9
*IKZF1*	7	50.3
*EGFR*	7	55.1
*BRAF*	7	140.4
*XRCC2*	7	152.3
*PAX5*	9	36.8
*TLR4*	9	120.5
*CYP2D6*	22	42.5
*HPRT1*	X	133.6

Abbreviation: AML, acute myeloid leukaemia.

**Table 2:** Diagnostic information and mutations in the dominant leukaemic clone of patient samples

*Sample ID*	*Age*	*Sex*	*Sample type*	*FAB*	*WCC (x10*^*9*^*/l)*	*BM blasts* %	*CD34*+%	*CD13*+%	*CD33*+%	*CD7*+%	*CD56*+%	*Karyotype*	*Mutations in dominant AML clone*			
P1	45	F	P	M5a	140	90	3	56	75	26	0	46XX	N*P*M1 L287fs*	*DNMT3A R882C*	*FLT3 D835Y*	
P2	71	M	P	M4	111	85	0	72	92	0	80	46XY	ASXL1 G642fs*	TET2 L1119*	*KRAS K117N*	*NF1 intron* 2
P3	73	M	P	M2	108	95	34	44	75	0	0	46XY	CEBPA A111fs*	*CEBPA T310NT*	WT1 R145fs*	*NRAS G12D*
P4	43	F	P	M1	24.4	95	0	12	81	0	2	46XX	NPM1 L287fs*	*IDH2 R140Q*		
P5	47	M	P	M5a	38	80	0	74	33	45	0	46XY	NPM1 L287fs*	*IDH1 R132H*	*FLT3 D835Y*	
P6	80	M	P	M1	116	95	0	53	99	6	79	46XY	ASXL1 C594*	TET2 P612fs*	*KRAS G12V*	
P7	59	F	P	M4	2.6	60	85	80	7	0	0	46XX	DNMT3A G590fs*	*IDH2 R172K*		
P1CR	45	F	CR	n/a	n/a	n/a	n/a	n/a	n/a	n/a	n/a	n/a				
P2CR	71	M	CR	n/a	n/a	n/a	n/a	n/a	n/a	n/a	n/a	n/a				
P4Rel	45	F	Rel1	M1	3	65%	nd	nd	nd	nd	nd	46XX	*NPM1c (TCTG)*	*IDH2 R140Q*		

Abbreviations: BM, bone marrow; CR, complete remission; F, female; M, male; n/a, not applicable; nd, not determined.

## Notes

Dr Bram Herman is an employee of Agilent Technologies, manufacturer of Sure Select cRNA baits used for targeted capture. His contribution to this work was limited to the bespoke design of our bait set to maximise target DNA capture and the provision of bait sequence files for bioinformatic analyses.
