# Supplementary material for: Detailed molecular characterisation of acute myeloid leukaemia with a normal karyotype using targeted DNA capture
Source: Leukemia. 2013 May 24;27(9):1820–5. doi: 10.1038/leu.2013.117 (PMC3768109; doi:10.1038/leu.2013.117)
Supplement: Supplementary Figure S1 [file leu2013117x1.ppt]

## Slide 1
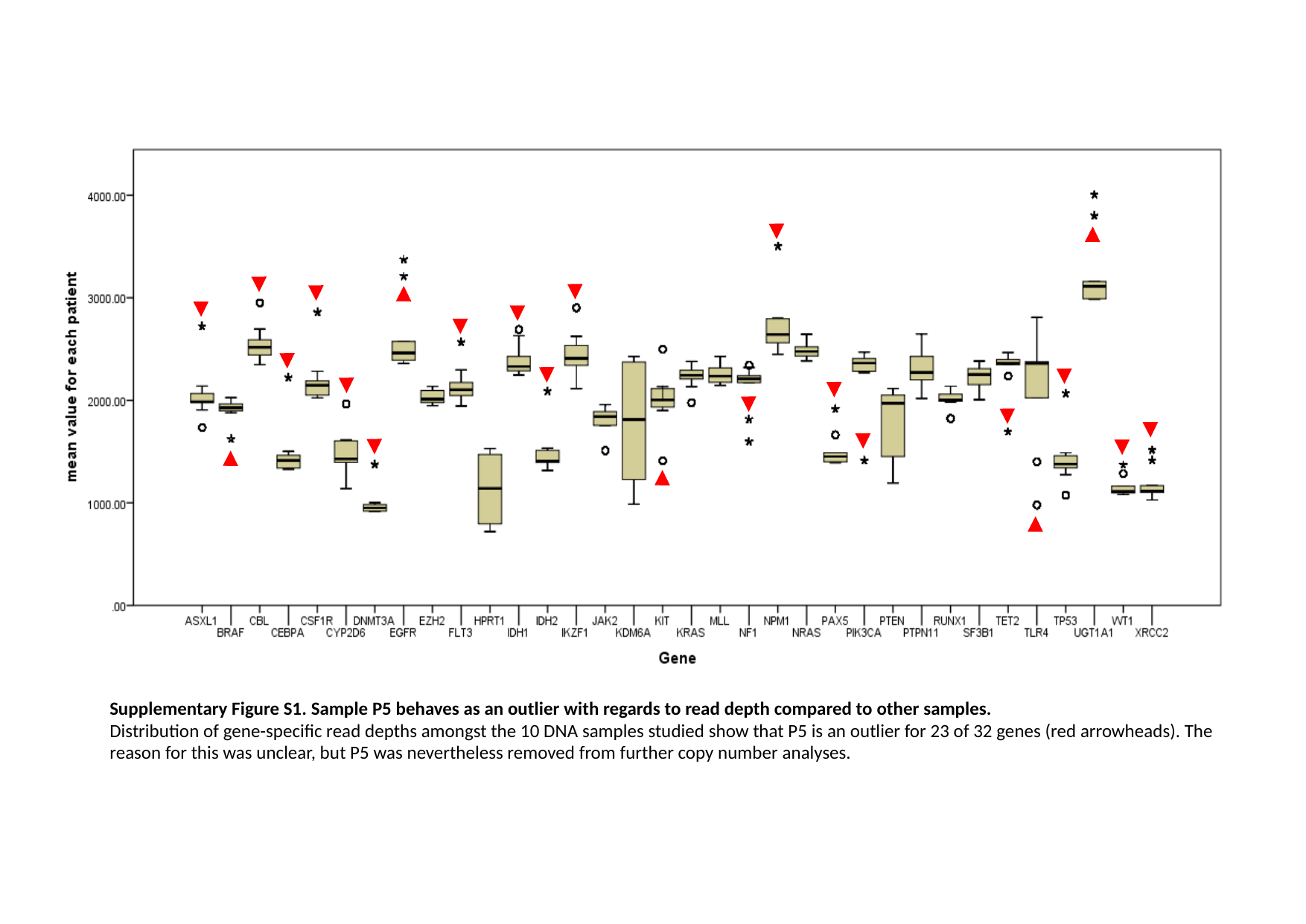

Supplementary Figure S1. Sample P5 behaves as an outlier with regards to read depth compared to other samples.
Distribution of gene-specific read depths amongst the 10 DNA samples studied show that P5 is an outlier for 23 of 32 genes (red arrowheads). The reason for this was unclear, but P5 was nevertheless removed from further copy number analyses.
