# Supplementary material for: Detailed molecular characterisation of acute myeloid leukaemia with a normal karyotype using targeted DNA capture
Source: Leukemia. 2013 May 24;27(9):1820–5. doi: 10.1038/leu.2013.117 (PMC3768109; doi:10.1038/leu.2013.117)
Supplement: Supplementary Figure S2 [file leu2013117x2.ppt]

## Slide 1
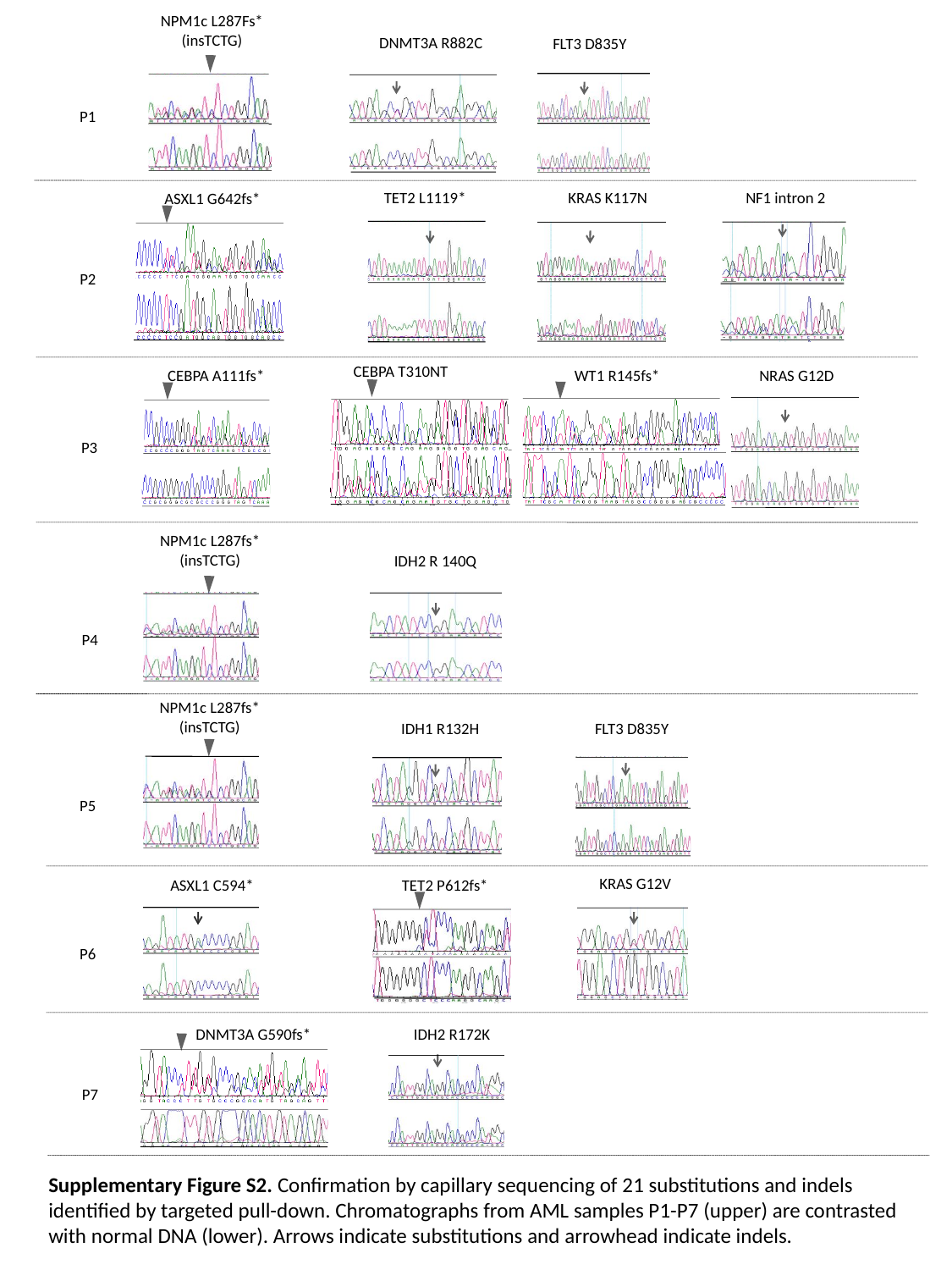

NPM1c L287Fs* (insTCTG)
DNMT3A R882C
FLT3 D835Y
P1
KRAS K117N
NF1 intron 2
TET2 L1119*
ASXL1 G642fs*
P2
CEBPA T310NT
P3
CEBPA A111fs*
NRAS G12D
WT1 R145fs*
P3
NPM1c L287fs*
(insTCTG)
IDH2 R 140Q
P4
NPM1c L287fs*
(insTCTG)
IDH1 R132H
FLT3 D835Y
P5
KRAS G12V
ASXL1 C594*
TET2 P612fs*
P6
DNMT3A G590fs*
IDH2 R172K
P7
Supplementary Figure S2. Confirmation by capillary sequencing of 21 substitutions and indels identified by targeted pull-down. Chromatographs from AML samples P1-P7 (upper) are contrasted with normal DNA (lower). Arrows indicate substitutions and arrowhead indicate indels.
