# Supplementary material for: Detailed molecular characterisation of acute myeloid leukaemia with a normal karyotype using targeted DNA capture
Source: Leukemia. 2013 May 24;27(9):1820–5. doi: 10.1038/leu.2013.117 (PMC3768109; doi:10.1038/leu.2013.117)
Supplement: Supplementary Figure S3 [file leu2013117x3.ppt]

## Slide 1
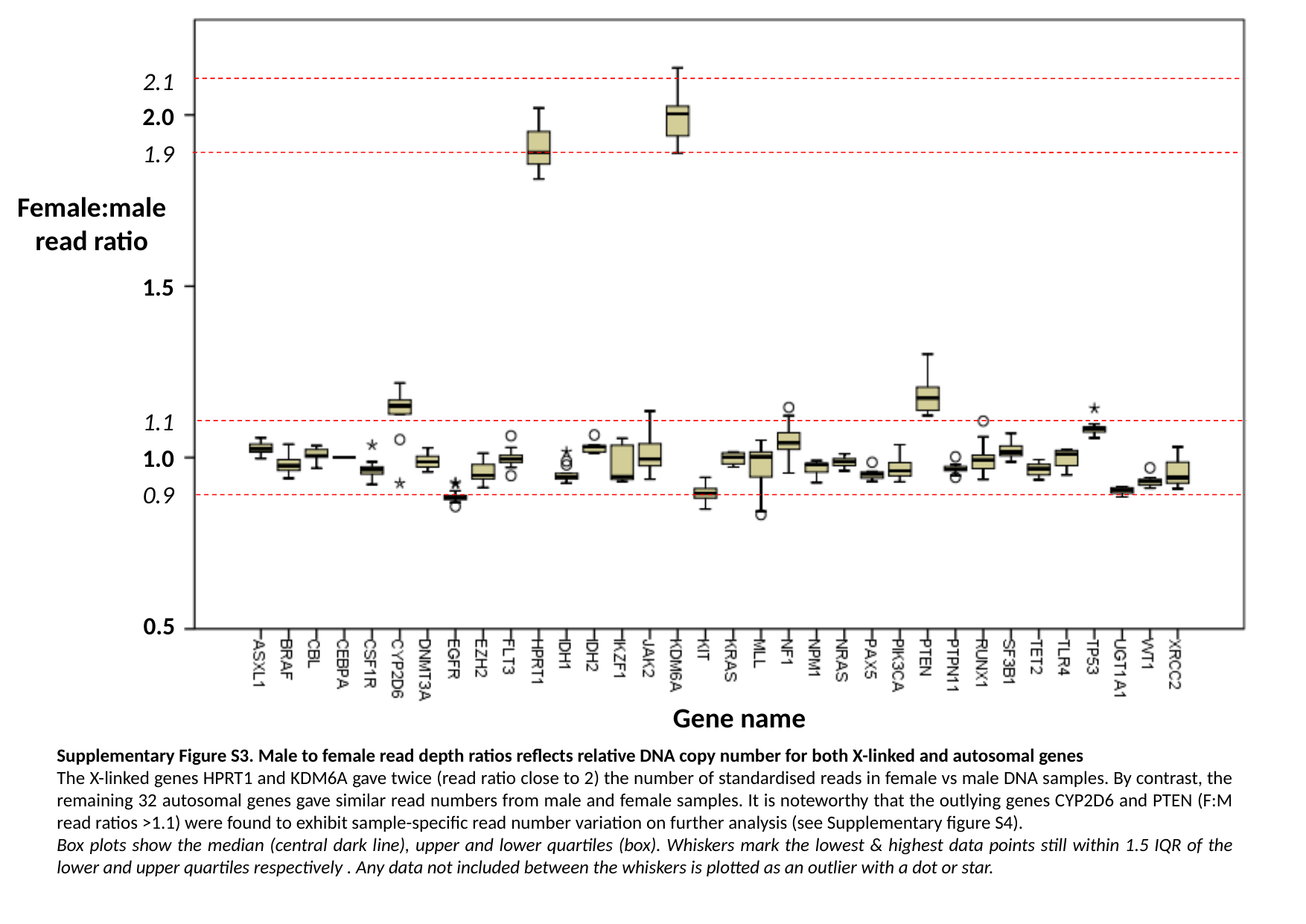

2.1
2.0
1.9
Female:male
read ratio
1.5
1.1
1.0
0.9
0.5
Gene name
Supplementary Figure S3. Male to female read depth ratios reflects relative DNA copy number for both X-linked and autosomal genes
The X-linked genes HPRT1 and KDM6A gave twice (read ratio close to 2) the number of standardised reads in female vs male DNA samples. By contrast, the remaining 32 autosomal genes gave similar read numbers from male and female samples. It is noteworthy that the outlying genes CYP2D6 and PTEN (F:M read ratios >1.1) were found to exhibit sample-specific read number variation on further analysis (see Supplementary figure S4).
Box plots show the median (central dark line), upper and lower quartiles (box). Whiskers mark the lowest & highest data points still within 1.5 IQR of the lower and upper quartiles respectively . Any data not included between the whiskers is plotted as an outlier with a dot or star.
