# Supplementary material for: Detailed molecular characterisation of acute myeloid leukaemia with a normal karyotype using targeted DNA capture
Source: Leukemia. 2013 May 24;27(9):1820–5. doi: 10.1038/leu.2013.117 (PMC3768109; doi:10.1038/leu.2013.117)
Supplement: Supplementary Figure S4 [file leu2013117x4.ppt]

## Slide 1
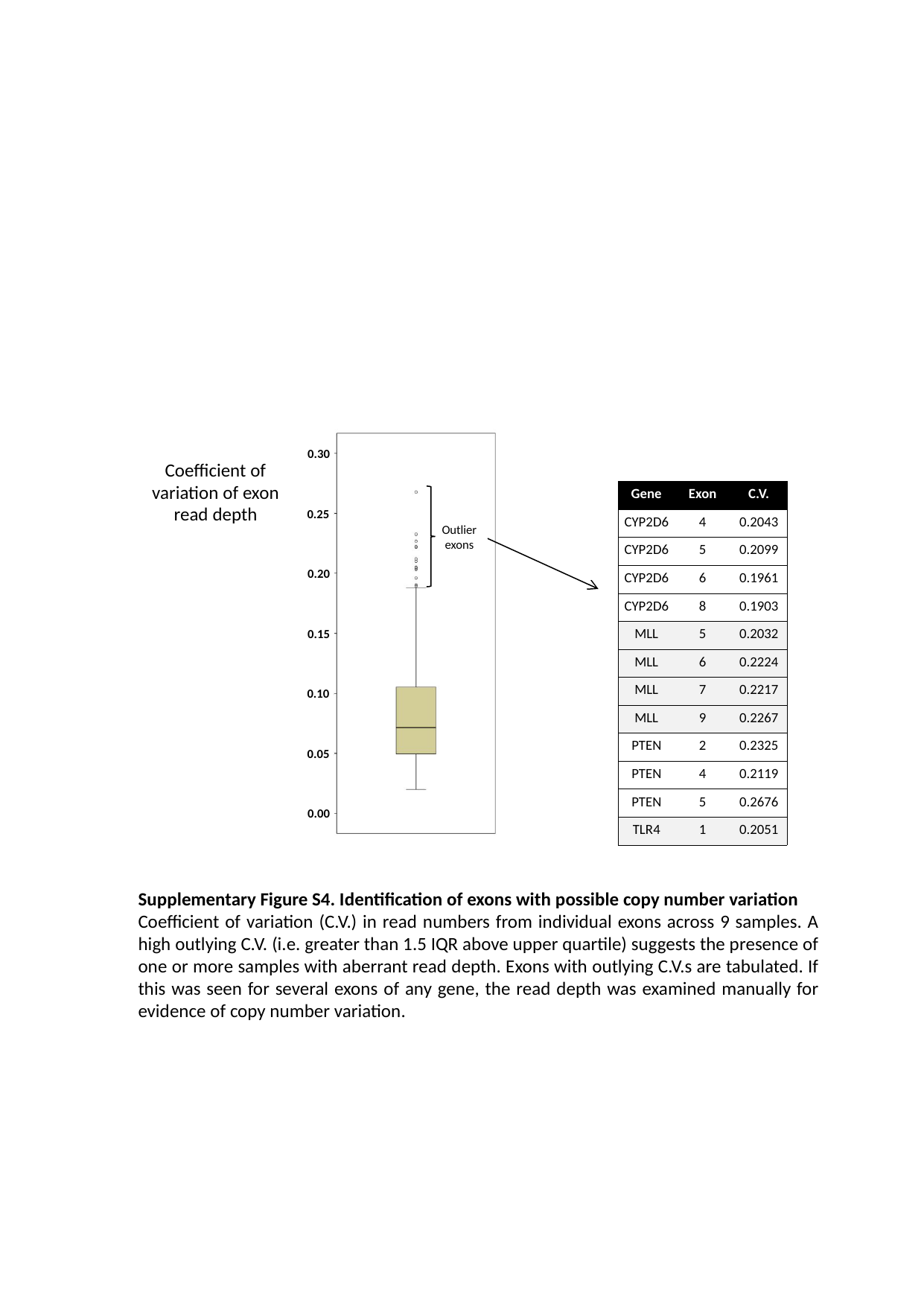

0.30
0.25
0.20
0.15
0.10
0.05
0.00
Coefficient of variation of exon read depth
| Gene | Exon | C.V. |
| --- | --- | --- |
| CYP2D6 | 4 | 0.2043 |
| CYP2D6 | 5 | 0.2099 |
| CYP2D6 | 6 | 0.1961 |
| CYP2D6 | 8 | 0.1903 |
| MLL | 5 | 0.2032 |
| MLL | 6 | 0.2224 |
| MLL | 7 | 0.2217 |
| MLL | 9 | 0.2267 |
| PTEN | 2 | 0.2325 |
| PTEN | 4 | 0.2119 |
| PTEN | 5 | 0.2676 |
| TLR4 | 1 | 0.2051 |
Outlier
exons
Supplementary Figure S4. Identification of exons with possible copy number variation
Coefficient of variation (C.V.) in read numbers from individual exons across 9 samples. A high outlying C.V. (i.e. greater than 1.5 IQR above upper quartile) suggests the presence of one or more samples with aberrant read depth. Exons with outlying C.V.s are tabulated. If this was seen for several exons of any gene, the read depth was examined manually for evidence of copy number variation.
