# Supplementary material for: Detailed molecular characterisation of acute myeloid leukaemia with a normal karyotype using targeted DNA capture
Source: Leukemia. 2013 May 24;27(9):1820–5. doi: 10.1038/leu.2013.117 (PMC3768109; doi:10.1038/leu.2013.117)
Supplement: Supplementary Figure S5 [file leu2013117x5.ppt]

## Slide 1
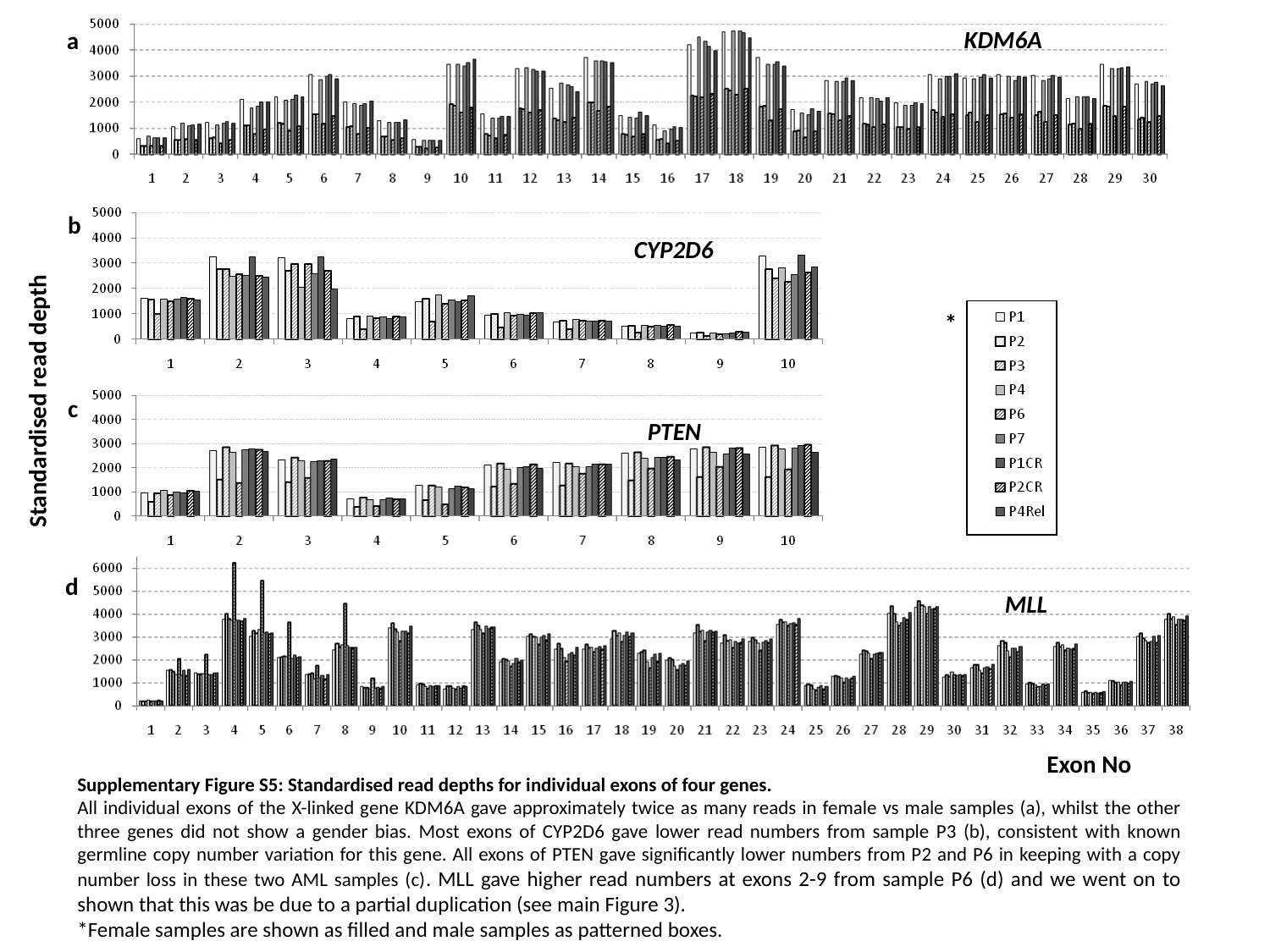

KDM6A
a
b
CYP2D6
*
Standardised read depth
c
PTEN
d
MLL
Exon No
Supplementary Figure S5: Standardised read depths for individual exons of four genes.
All individual exons of the X-linked gene KDM6A gave approximately twice as many reads in female vs male samples (a), whilst the other three genes did not show a gender bias. Most exons of CYP2D6 gave lower read numbers from sample P3 (b), consistent with known germline copy number variation for this gene. All exons of PTEN gave significantly lower numbers from P2 and P6 in keeping with a copy number loss in these two AML samples (c). MLL gave higher read numbers at exons 2-9 from sample P6 (d) and we went on to shown that this was be due to a partial duplication (see main Figure 3).
*Female samples are shown as filled and male samples as patterned boxes.
