# Supplementary material for: Detailed molecular characterisation of acute myeloid leukaemia with a normal karyotype using targeted DNA capture
Source: Leukemia. 2013 May 24;27(9):1820–5. doi: 10.1038/leu.2013.117 (PMC3768109; doi:10.1038/leu.2013.117)
Supplement: Supplementary Figure S6 [file leu2013117x6.ppt]

## Slide 1
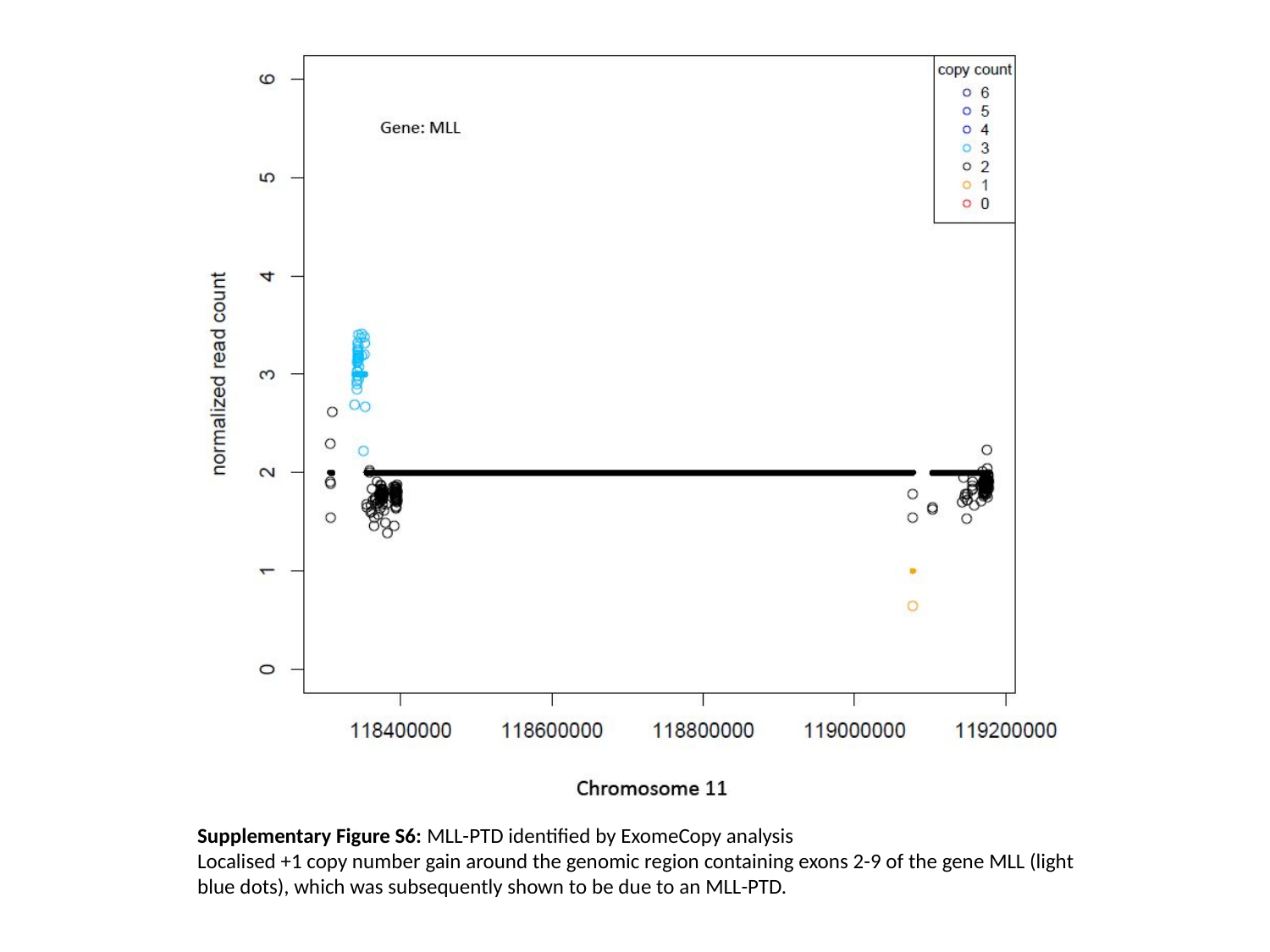

Supplementary Figure S6: MLL-PTD identified by ExomeCopy analysis
Localised +1 copy number gain around the genomic region containing exons 2-9 of the gene MLL (light blue dots), which was subsequently shown to be due to an MLL-PTD.
