# Supplementary material for: Detailed molecular characterisation of acute myeloid leukaemia with a normal karyotype using targeted DNA capture
Source: Leukemia. 2013 May 24;27(9):1820–5. doi: 10.1038/leu.2013.117 (PMC3768109; doi:10.1038/leu.2013.117)
Supplement: Supplementary Table S1 [file leu2013117x7.pdf]

Primer name	Sequence
ASXL1 C594*_F	TCACACAGTCCCACCAGAAA
ASXL1 C594*_R	CAGAGCACGGGCTTTAATGT
ASXL1_G642fs*_F	GAGGTCACCACTGCCATAGA
ASXL1_G642fs*_R	TACACTTTCCAGGGGTGCTC
CEBPA_A111fs*_F	CTTCA ACGA CGAGTTCCTGGCCGA
CEBPA_A111fs*_R	AGCTGCTTGGCTTCATCCTCCT
CEBPA_T310NT_F	TTGTCACTGGTCAGCTCCAG
CEBPA_T310NT_R	TGGACAAGAACAGCAACGAG
DNMT3A_R882C_F	CCATGTCCCTTACACACACG
DNMT3A_R882C_R	CTGAGTGCCGGGTTGTTTAT
DNMT3A_G590fs*_F	CCCAGCACTCACAAATTCCT
DNMT3A_G590fs*_R	CAGGCAGCCATTAAGGAAGA
FLT3_D835Y_F	CCTGAAGCTGCAGAAAAACC
FLT3_D835Y_R	TCCATCACCGGTACCTCCTA
IDH1_R132H_F	ATTGTGCAGCCAGTGTTGAA
IDH1_R132H_R	CGGTCTTCAGAGAAGCCATT
IDH2_R140Q_F	TGTGGCCTTGTACTGCAGAG
IDH2_R140Q_R	GACTCCAGAGCCCACACATT
IDH2_R172K_F	TGTGGCCTTGTACTGCAGAG
IDH2_R172K_R	GACTCCAGAGCCCACACATT
KRAS_G12V_F	AGAATGGTCCTGCACCAGTAA
KRAS_G12V_R	CTTAAGCGTCGATGGAGGAG
KRAS_K117N_F	AGAAACCAAAGCCAAAAGCA
KRAS_K117N_R	TTGTGGACAGGTTTTGAAAGA
NF1_intron_F	TGCCATTTCTGTTTGCCTTA
NF1_intron_R	TTCTGCTACCAATCAACACACA
NPM1_ex12_F	ATTGGCCATATGGGTCTCTG
NPM1_ex12_R	AACACGGTAGGGAAAGTTCTCA
NRAS_G12D_F	CACTGGGCCTCACCTCTATG
NRAS_G12D_R	GCCCAAGGACTGTTGAAAAA
TET2_L119*_F	GTTGAAATGTCAGGGCCAGT
TET2_L119*_R	CAAATTGCTGCCAGACTCAA
TET2_P612fs*_F	GAGGCATCACTGCCATCAAT
TET2_P612fs*_R	TGCTCCAGCTGTGTTGTTTT
WT1_GATK_AML3_F	TGTCCTAGAGCGGAGAGTCC
WT1_GATK_AML3_+140R	CAGGCCAGGATGTTTCCTAA
FLT3-ITD_F	GCAATTTAGGTATGAAAGCCAGC
FLT3-ITD_R	CTTTCAGCATTTTGACGGCAACC
MLL_PTD_F	TCCAGAGCAGAGCAAACAGA
MLL_PTD_R	TATGTGGCCTTTGGTTCTCC
MiSeq_rs2736627_F1	**ACACTCTTTCCCTACACGACGCTCTTCCGATCT**CAGCTGATGAAGAACTTGCTTG
MiSeq_rs2736627_R1	**TCGGCATTCCTGCTGAACCGCTCTTCCGATCT**ACCTTTGCCCCAGATTTCAG
MiSeq_rs701848_F2	**ACACTCTTTCCCTACACGACGCTCTTCCGATCT**CCTACATGTGCTTTATTGATTTGC
MiSeq_rs701848_R2	**TCGGCATTCCTGCTGAACCGCTCTTCCGATCT**CCGCTTAAAATCGTATGCAGT
MiSeq_rs1234213_F1	**ACACTCTTTCCCTACACGACGCTCTTCCGATCT**CCAAAGAAAGTGTAAAATGTGACTG
MiSeq_rs1234213_R1	**TCGGCATTCCTGCTGAACCGCTCTTCCGATCT**TCAAAACAAAACAAGCTTTTCC
MiSeq_rs1234224_F1	**ACACTCTTTCCCTACACGACGCTCTTCCGATCT**TGCAAGTGTCGGGAAGTGTA
MiSeq_rs1234224_R1	**TCGGCATTCCTGCTGAACCGCTCTTCCGATCT**CCTGAGGGATTAGGGAAGGA
MiSeq_rs74146236_F2	**TCGGCATTCCTGCTGAACCGCTCTTCCGATCT**TGGTTTGACAGAGGGGAAAC
MiSeq_rs74146236_R2	**TCGGCATTCCTGCTGAACCGCTCTTCCGATCT**CCTGCAGATGCAAAATTCCT
MiSeq_rs3895069_F1	**ACACTCTTTCCCTACACGACGCTCTTCCGATCT**TGGCAATAGGACATTGTGTCA
MiSeq_rs3895069_R1	**TCGGCATTCCTGCTGAACCGCTCTTCCGATCT**TAAAACGGGAAAGTGCCATC
MiSeq_tag_F	AATGATACGGCGACCACCGAGATCTACACTCTTTCCCTACACGACGCTCTTCCGATC*T
iPCRtagR1	CAAGCAGAAGACGGCATACGAGATAACGTGATGAGATCGGTCTCGGCATTCCTGCTGAACCGCTCTTCCGATC*T
iPCRtagR2	CAAGCAGAAGACGGCATACGAGATAAACATCGGAGATCGGTCTCGGCATTCCTGCTGAACCGCTCTTCCGATC*T
iPCRtagR3	CAAGCAGAAGACGGCATACGAGATATGCCTAAGAGATCGGTCTCGGCATTCCTGCTGAACCGCTCTTCCGATC*T
iPCRtagR4	CAAGCAGAAGACGGCATACGAGATAGTGGTCAGAGATCGGTCTCGGCATTCCTGCTGAACCGCTCTTCCGATC*T
iPCRtagR5	CAAGCAGAAGACGGCATACGAGATACCACTGTGAGATCGGTCTCGGCATTCCTGCTGAACCGCTCTTCCGATC*T
iPCRtagR6	CAAGCAGAAGACGGCATACGAGATACATTGGCGAGATCGGTCTCGGCATTCCTGCTGAACCGCTCTTCCGATC*T
iPCRtagR7	CAAGCAGAAGACGGCATACGAGATCAGATCTGGAGATCGGTCTCGGCATTCCTGCTGAACCGCTCTTCCGATC*T
iPCRtagR8	CAAGCAGAAGACGGCATACGAGATCATCAAGTGAGATCGGTCTCGGCATTCCTGCTGAACCGCTCTTCCGATC*T
iPCRtagR9	CAAGCAGAAGACGGCATACGAGATCGCTGATCGAGATCGGTCTCGGCATTCCTGCTGAACCGCTCTTCCGATC*T
iPCRtagR10	CAAGCAGAAGACGGCATACGAGATACAAGCTAGAGATCGGTCTCGGCATTCCTGCTGAACCGCTCTTCCGATC*T
MiSeq_sequencing_oligo	AAGAGCGGTTCAGCAGGAATGCCGAGACCGATCTC

Supplementary Table S1. Oligonucletide primers used for validation of mutations

Primers names reflect the mutations or SNPs they were used to test/validate
