# Supplementary material for: Detailed molecular characterisation of acute myeloid leukaemia with a normal karyotype using targeted DNA capture
Source: Leukemia. 2013 May 24;27(9):1820–5. doi: 10.1038/leu.2013.117 (PMC3768109; doi:10.1038/leu.2013.117)
Supplement: Supplementary Table S3 [file leu2013117x9.pdf]

Mutation Caller	Sample	Chromosome	Position	WtAllele	MutAllele	SomaticScore	RefReads Normal	VariantReads Normal	Variant FreqNormal	RefReads Tumor	Variant Reads Tumor	Variant Freq Tumor	Gene_ID	Transcript_ID	c.Annot	p.Annot	result	ratio	Validated? -method
MIDAS	P3	Chr1	115258747	C	T	0	3724	1	0.000268456	3060	1492	0.3270	NRAS	ENST00000369535_r69	c.35G>A	p.G12D	MISSENSE	(1\1)	Yes - Sanger
SS	P3	Chr1	115258747	C	T	225	3724	0	0	3060	1538	0.3334	NRAS	ENST00000369535_r69	c.35G>A	p.G12D	MISSENSE	(1\1)	Yes - Sanger
VS_D	P3	Chr1	115258747	C	T	0	3710	1	0.000269469	3045	1523	0.3334	NRAS	ENST00000369535_r69	c.35G>A	p.G12D	MISSENSE	(1\1)	Yes - Sanger
VS_HS	P3	Chr1	115258747	C	T	0	3710	1	0.000269469	3045	1523	0.3334	NRAS	ENST00000369535_r69	c.35G>A	p.G12D	MISSENSE	(1\1)	Yes - Sanger
MIDAS	P1	Chr2	25457243	G	A	0	1570	0	0	1001	796	0.4430	DNMT3A	ENST00000380746_r69	c.2077C>T	p.R693C (R882C)	MISSENSE	(3\3)	Yes - Sanger
SS	P1	Chr2	25457243	G	A	225	1570	0	0	1001	831	0.4534	DNMT3A	ENST00000380746_r69	c.2077C>T	p.R693C (R882C)	MISSENSE	(3\3)	Yes - Sanger
VS_D	P1	Chr2	25457243	G	A	2.667547187685072E-269	1562	0	0	993	813	0.4502	DNMT3A	ENST00000380746_r69	c.2077C>T	p.R693C (R882C)	MISSENSE	(3\3)	Yes - Sanger
VS_HS	P1	Chr2	25457243	G	A	2.667547187685072E-269	1562	0	0	993	813	0.4502	DNMT3A	ENST00000380746_r69	c.2077C>T	p.R693C (R882C)	MISSENSE	(3\3)	Yes - Sanger
MIDAS	P7	Chr2	25467106	C	-	0	0	0	0	1014	435	0.3002	DNMT3A	ENST00000380746_r69	c.1202G>-	p.G401fs*61 (G590fs*)	FRAMESHIFT		Yes - Sanger
VS_D	P7	Chr2	25467105	C	-	2.6350030933505124E-151	1413	0	0	951	435	0.3139	DNMT3A	ENST00000380746_r69	c.1203G>-	p.G401fs*61 (G590fs*)	FRAMESHIFT	(3\3)	Yes - Sanger
VS_HS	P7	Chr2	25467105	C	-	2.6350030933505124E-151	1413	0	0	951	435	0.3139	DNMT3A	ENST00000380746_r69	c.1203G>-	p.G401fs*61 (G590fs*)	FRAMESHIFT	(3\3)	Yes - Sanger
MIDAS	P5	Chr2	209113112	C	T	0	3916	1	0.000510334	3274	2469	0.4298	IDH1	ENST00000345146_r69	c.395G>A	p.R132H	MISSENSE	(3\3)	Yes - Sanger
SS	P5	Chr2	209113112	C	T	225	3917	0	0	3274	2530	0.4356	IDH1	ENST00000345146_r69	c.395G>A	p.R132H	MISSENSE	(3\3)	Yes - Sanger
VS_D	P5	Chr2	209113112	C	T	0	3894	1	0.000256739	3243	2511	0.4364	IDH1	ENST00000345146_r69	c.395G>A	p.R132H	MISSENSE	(3\3)	Yes - Sanger
VS_HS	P5	Chr2	209113112	C	T	0	3894	1	0.000256739	3243	2511	0.4364	IDH1	ENST00000345146_r69	c.395G>A	p.R132H	MISSENSE	(3\3)	Yes - Sanger
MIDAS	P2	Chr4	106158455	T	G	0	3366	0	0	2247	1865	0.4536	TET2	ENST00000305737_r69	c.3356T>G	p.L1119*	NONSENSE	(4\4)	Yes - Sanger
SS	P2	Chr4	106158455	T	G	225	3366	0	0	2247	1902	0.4583	TET2	ENST00000305737_r69	c.3356T>G	p.L1119*	NONSENSE	(4\4)	Yes - Sanger
VS_D	P2	Chr4	106158455	T	G	0	3362	0	0	2243	1888	0.4570	TET2	ENST00000305737_r69	c.3356T>G	p.L1119*	NONSENSE	(4\4)	Yes - Sanger
VS_HS	P2	Chr4	106158455	T	G	0	3362	0	0	2243	1888	0.4570	TET2	ENST00000305737_r69	c.3356T>G	p.L1119*	NONSENSE	(4\4)	Yes - Sanger
MIDAS	P1	Chr5	170837544	-	TCTG	0	0	1	0	1449	616	0.2983	NPM1	ENST00000517671_r69	c.860->TCTG	p.L287fs*13	FRAMESHIFT		Yes - Sanger
VS_D	P1	Chr5	170837543	-	TCTG	6.582920250438554E-227	2203	1	0.000453721	1329	614	0.3160	NPM1	ENST00000517671_r69	c.859->TCTG	p.L287fs*13	FRAMESHIFT	(3\3)	Yes - Sanger
VS_HS	P1	Chr5	170837543	-	TCTG	6.582920250438554E-227	2203	1	0.000453721	1329	614	0.3160	NPM1	ENST00000517671_r69	c.859->TCTG	p.L287fs*13	FRAMESHIFT	(3\3)	Yes - Sanger
MIDAS	P4	Chr5	170837544	-	TCTG	0	0	1	0	1180	456	0.2787	NPM1	ENST00000517671_r69	c.860->TCTG	p.L287fs*13	FRAMESHIFT		Yes - Sanger
VS_D	P4	Chr5	170837543	-	TCTG	6.761909647462354E-194	2203	1	0.000453721	1088	456	0.2953	NPM1	ENST00000517671_r69	c.859->TCTG	p.L287fs*13	FRAMESHIFT	(3\3)	Yes - Sanger
VS_HS	P4	Chr5	170837543	-	TCTG	6.761909647462354E-194	2203	1	0.000453721	1088	456	0.2953	NPM1	ENST00000517671_r69	c.859->TCTG	p.L287fs*13	FRAMESHIFT	(3\3)	Yes - Sanger
MIDAS	P4Rel	Chr5	170837544	-	TCTG	0	0	1	0	1912	401	0.1734	NPM1 ‡	ENST00000517671_r69	c.860->TCTG	p.L287fs*13	FRAMESHIFT		Yes - Sanger
VS_HS	P4Rel	Chr5	170837543	-	TCTG	3.733283817427552E-127	2203	1	0.000453721	1804	399	0.1811	NPM1 ‡	ENST00000517671_r69	c.859->TCTG	p.L287fs*13	FRAMESHIFT	(3\3)	Yes - Sanger
VS_D	P5	Chr5	170837543	-	TCTG	3.300468663291457E-246	2203	1	0.000453721	1829	830	0.3121	NPM1	ENST00000517671_r69	c.859->TCTG	p.L287fs*13	FRAMESHIFT	(3\3)	Yes - Sanger
VS_HS	P5	Chr5	170837543	-	TCTG	3.300468663291457E-246	2203	1	0.000453721	1829	830	0.3121	NPM1	ENST00000517671_r69	c.859->TCTG	p.L287fs*13	FRAMESHIFT	(3\3)	Yes - Sanger
MIDAS	P5	Chr5	170837544	-	TCTG	0	0	1	0	1986	836	0.2962	NPM1	ENST00000517671_r69	c.860->TCTG	p.L287fs*13	FRAMESHIFT		Yes - Sanger
MIDAS	P3	Chr11	32456253	-	GCGAATA	0	0	0	0	302	87	0.2237	WT1	ENST00000332351_r69	c.639->TATTCGC	p.R213fs*37	FRAMESHIFT		Yes - Sanger
VS_D	P3	Chr11	32456252	-	GCGAATA	3.80673628861356E-30	405	0	0	301	87	0.2242	WT1	ENST00000332351_r69	c.640->TATTCGC	p.N214fs*36	FRAMESHIFT	(3\3)	Yes - Sanger
VS_HS	P3	Chr11	32456252	-	GCGAATA	3.80673628861356E-30	405	0	0	301	87	0.2242	WT1	ENST00000332351_r69	c.640->TATTCGC	p.N214fs*36	FRAMESHIFT	(3\3)	Yes - Sanger
MIDAS	P4	Chr11	32456486	G	A	0	333	0	0	152	98	0.3920	WT1	ENST00000332351_r69	c.406C>T	p.P136S	MISSENSE	(3\3)	Assumed germline
SS	P4	Chr11	32456486	G	A	210	333	0	0	152	150	0.4934	WT1	ENST00000332351_r69	c.406C>T	p.P136S	MISSENSE	(3\3)	Assumed germline
VS_D	P4	Chr11	32456486	G	A	1.074039345730627E-54	320	0	0	145	133	0.4784	WT1	ENST00000332351_r69	c.406C>T	p.P136S	MISSENSE	(3\3)	Assumed germline
VS_HS	P4	Chr11	32456486	G	A	1.074039345730627E-54	320	0	0	145	133	0.4784	WT1	ENST00000332351_r69	c.406C>T	p.P136S	MISSENSE	(3\3)	Assumed germline
MIDAS	P4Rel	Chr11	32456486	G	A	0	333	0	0	160	99	0.3822	WT1	ENST00000332351_r69	c.406C>T	p.P136S	MISSENSE	(3\3)	Assumed germline
SS	P4Rel	Chr11	32456486	G	A	225	333	0	0	160	166	0.5030	WT1	ENST00000332351_r69	c.406C>T	p.P136S	MISSENSE	(3\3)	Assumed germline
VS_D	P4Rel	Chr11	32456486	G	A	1.0889005961944242E-56	320	0	0	156	145	0.4817	WT1	ENST00000332351_r69	c.406C>T	p.P136S	MISSENSE	(3\3)	Assumed germline
VS_HS	P4Rel	Chr11	32456486	G	A	1.0889005961944242E-56	320	0	0	156	145	0.4817	WT1	ENST00000332351_r69	c.406C>T	p.P136S	MISSENSE	(3\3)	Assumed germline
MIDAS	P2	Chr12	25378647	T	A	0	2771	0	0	1954	1437	0.4238	KRAS	ENST00000311936_r69	c.351A>T	p.K117N	MISSENSE	(2\2)	Yes - Sanger
SS	P2	Chr12	25378647	T	A	225	2771	0	0	1954	1449	0.4258	KRAS	ENST00000311936_r69	c.351A>T	p.K117N	MISSENSE	(2\2)	Yes - Sanger
VS_D	P2	Chr12	25378647	T	A	0	2767	1	0.000361272	1943	1445	0.4265	KRAS	ENST00000311936_r69	c.351A>T	p.K117N	MISSENSE	(2\2)	Yes - Sanger
VS_HS	P2	Chr12	25378647	T	A	0	2767	1	0.000361272	1943	1445	0.4265	KRAS	ENST00000311936_r69	c.351A>T	p.K117N	MISSENSE	(2\2)	Yes - Sanger
MIDAS	P6	Chr12	25398284	C	A	0	2619	0	0.000381679	1326	742	0.3588	KRAS	ENST00000311936_r69	c.35G>T	p.G12V	MISSENSE	(2\2)	Yes - Sanger
SS	P6	Chr12	25398284	C	A	225	2619	0	0	1326	760	0.3638	KRAS	ENST00000311936_r69	c.35G>T	p.G12V	MISSENSE	(2\2)	Yes - Sanger
VS_D	P6	Chr12	25398284	C	A	6.820621128995054E-305	2596	1	0.00038506	1309	752	0.3649	KRAS	ENST00000311936_r69	c.35G>T	p.G12V	MISSENSE	(2\2)	Yes - Sanger
VS_HS	P6	Chr12	25398284	C	A	6.820621128995054E-305	2596	1	0.00038506	1309	752	0.3649	KRAS	ENST00000311936_r69	c.35G>T	p.G12V	MISSENSE	(2\2)	Yes - Sanger
MIDAS	P1	Chr13	28592642	C	A	0	2708	0	0	1979	1217	0.3808	FLT3	ENST00000241453_r69	c.2503G>T	p.D835Y	MISSENSE	(1\1)	Yes - Sanger
SS	P1	Chr13	28592642	C	A	225	2708	0	0	1981	1305	0.3974	FLT3	ENST00000241453_r69	c.2503G>T	p.D835Y	MISSENSE	(1\1)	Yes - Sanger
VS_D	P1	Chr13	28592642	C	A	0	2672	0	0	1949	1272	0.3949	FLT3	ENST00000241453_r69	c.2503G>T	p.D835Y	MISSENSE	(1\1)	Yes - Sanger
VS_HS	P1	Chr13	28592642	C	A	0	2672	0	0	1949	1272	0.3949	FLT3	ENST00000241453_r69	c.2503G>T	p.D835Y	MISSENSE	(1\1)	Yes - Sanger
MIDAS	P5	Chr13	28592642	C	A	0	2708	0	0	3370	1432	0.2982	FLT3	ENST00000241453_r69	c.2503G>T	p.D835Y	MISSENSE	(1\1)	Yes - Sanger
SS	P5	Chr13	28592642	C	A	225	2708	0	0	3370	1547	0.3144	FLT3	ENST00000241453_r69	c.2503G>T	p.D835Y	MISSENSE	(1\1)	Yes - Sanger
VS_D	P5	Chr13	28592642	C	A	0	2672	0	0	3289	1512	0.3149	FLT3	ENST00000241453_r69	c.2503G>T	p.D835Y	MISSENSE	(1\1)	Yes - Sanger
VS_HS	P5	Chr13	28592642	C	A	0	2672	0	0	3289	1512	0.3149	FLT3	ENST00000241453_r69	c.2503G>T	p.D835Y	MISSENSE	(1\1)	Yes - Sanger
SS	P5	Chr15	90628533	T	G	7	43	0	0	47	31	0.3974	IDH2	ENST00000330062_r69	c.1054A>C	p.T352P	MISSENSE	(1\1)	**No (MiSeq)**
MIDAS	P7	Chr15	90631838	C	T	0	1663	1	0.000600962	1301	549	0.2968	IDH2	ENST00000330062_r69	c.515G>A	p.R172K	MISSENSE	(1\1)	Yes - Sanger
SS	P7	Chr15	90631838	C	T	225	1663	0	0	1301	563	0.3019	IDH2	ENST00000330062_r69	c.515G>A	p.R172K	MISSENSE	(1\1)	Yes - Sanger
VS_D	P7	Chr15	90631838	C	T	4.825704787591387E-174	1657	1	0.000603136	1292	559	0.3020	IDH2	ENST00000330062_r69	c.515G>A	p.R172K	MISSENSE	(1\1)	Yes - Sanger
VS_HS	P7	Chr15	90631838	C	T	4.825704787591387E-174	1657	1	0.000603136	1292	559	0.3020	IDH2	ENST00000330062_r69	c.515G>A	p.R172K	MISSENSE	(1\1)	Yes - Sanger
MIDAS	P4	Chr15	90631934	C	T	0	2007	1	0.000498008	1092	750	0.4072	IDH2	ENST00000330062_r69	c.419G>A	p.R140Q	MISSENSE	(1\1)	Yes - Sanger
SS	P4	Chr15	90631934	C	T	225	2007	0	0	1092	764	0.4116	IDH2	ENST00000330062_r69	c.419G>A	p.R140Q	MISSENSE	(1\1)	Yes - Sanger
VS_D	P4	Chr15	90631934	C	T	3.6622571770368665E-286	1997	1	0.000500501	1086	764	0.4130	IDH2	ENST00000330062_r69	c.419G>A	p.R140Q	MISSENSE	(1\1)	Yes - Sanger
VS_HS	P4	Chr15	90631934	C	T	3.6622571770368665E-286	1997	1	0.000500501	1086	764	0.4130	IDH2	ENST00000330062_r69	c.419G>A	p.R140Q	MISSENSE	(1\1)	Yes - Sanger
MIDAS	P4Rel	Chr15	90631934	C	T	0	2007	1	0.000498008	1732	610	0.2603	IDH2	ENST00000330062_r69	c.419G>A	p.R140Q	MISSENSE	(1\1)	Yes - Sanger
SS	P4Rel	Chr15	90631934	C	T	225	2007	0	0	1730	622	0.2641	IDH2	ENST00000330062_r69	c.419G>A	p.R140Q	MISSENSE	(1\1)	Yes - Sanger
VS_D	P4Rel	Chr15	90631934	C	T	1.5726764241508648E-182	1997	1	0.000500501	1722	617	0.2638	IDH2	ENST00000330062_r69	c.419G>A	p.R140Q	MISSENSE	(1\1)	Yes - Sanger
VS_HS	P4Rel	Chr15	90631934	C	T	1.5726764241508648E-182	1997	1	0.000500501	1722	617	0.2638	IDH2	ENST00000330062_r69	c.419G>A	p.R140Q	MISSENSE	(1\1)	Yes - Sanger
SS	P5	Chr17	7572980	T	G	19	1018	0	0	1771	460	0.2058	TP53	ENST00000269305_r69	c.1129A>C	p.T377P	MISSENSE	(2\2)	**No (Miseq)**
MIDAS	P3	Chr19	33792393	-	TGT	0	0	0	0	1601	1061	0.3986	CEBPA	ENST00000498907_r69	c.928->ACA	p.T310Ins(T)	MISSENSE		Yes - Sanger
VS_D	P3	Chr19	33792392	-	TGT	0	2387	1	0.000418761	1479	1055	0.4163	CEBPA	ENST00000498907_r69	c.929->ACA	p.T310T>NT	MISSENSE	(1\1)	Yes - Sanger
VS_HS	P3	Chr19	33792392	-	TGT	0	2387	1	0.000418761	1479	1055	0.4163	CEBPA	ENST00000498907_r69	c.929->ACA	p.T310T>NT	MISSENSE	(1\1)	Yes - Sanger
MIDAS	P3	Chr19	33792982	CGCGGGCG	-	0	0	0	0	21	35	0.6250	CEBPA	ENST00000498907_r69	c.332CGCCCGCG>-	p.A111fs*56	FRAMESHIFT		Yes - Sanger
VS_D	P3	Chr19	33792981	CGCGGGCG	-	2.1251223597062944E-16	67	0	0	21	35	0.6250	CEBPA	ENST00000498907_r69	c.333CGCCCGCG>-	p.A111fs*56	FRAMESHIFT	(1\1)	Yes - Sanger
VS_HS	P3	Chr19	33792981	CGCGGGCG	-	2.1251223597062944E-16	67	0	0	21	35	0.6250	CEBPA	ENST00000498907_r69	c.333CGCCCGCG>-	p.A111fs*56	FRAMESHIFT	(1\1)	Yes - Sanger
MIDAS	P6	Chr20	31022297	C	A	0	1793	1	0.000557103	745	428	0.3646	ASXL1	ENST00000375687_r69	c.1782C>A	p.C594*	NONSENSE	(1\1)	Yes - Sanger
SS	P6	Chr20	31022297	C	A	225	1793	0	0	745	559	0.4280	ASXL1	ENST00000375687_r69	c.1782C>A	p.C594*	NONSENSE	(1\1)	Yes - Sanger
VS_D	P6	Chr20	31022297	C	A	2.2107791799557503E-236	1771	1	0.000564334	727	530	0.4216	ASXL1	ENST00000375687_r69	c.1782C>A	p.C594*	NONSENSE	(1\1)	Yes - Sanger
VS_HS	P6	Chr20	31022297	C	A	2.2107791799557503E-236	1771	1	0.000564334	727	530	0.4216	ASXL1	ENST00000375687_r69	c.1782C>A	p.C594*	NONSENSE	(1\1)	Yes - Sanger
MIDAS †	P6	Chr4	106156936	-	G	0	10454	4†	0.000382482	974	3174	0.7652	TET2	ENST00000305737_r69	c.1837 ->G	p.G613fs*25	FRAMESHIFT	(4\4)	Yes-Sanger
MIDAS †	P2	Chr20	31022442	-	G	0	1404	3†	0.002132196	613	224	0.2676	ASXL1	ENST00000375687_r69	c.1927 ->G	p.G643fs*15	FRAMESHIFT	(1\1)	Yes - Sanger

Supplementary Table S3: Substitutions and indels identified in >20% of reads by MIDAS and another 3 mutation callers using sample P1CR as the reference control

† These mutations were not identified by VarScan because of >2 variant reads were coincidentally founds in the control sample (P1CR). They were identified by VarScan but not Somatic Sniper when a different control sample was used

‡ Variant in thie relapsed sample is included despite representing <20% reads as mutation was identified in the diagnostic sample

Known SNPs and synonymous substitutions were removed. MIDAS=Mutation Identification and Analysis Software, SS=Somatic Sniper, VS_D=VarScan Default, VS_HS=Varscan High Sensitivity
