# Supplementary material for: Detailed molecular characterisation of acute myeloid leukaemia with a normal karyotype using targeted DNA capture
Source: Leukemia. 2013 May 24;27(9):1820–5. doi: 10.1038/leu.2013.117 (PMC3768109; doi:10.1038/leu.2013.117)
Supplement: Supplementary Table S4 [file leu2013117x10.pdf]

SNP_ID	Sample	Chromosome	Position	Allele 1	Allele 2	Allele1_reads	Allele2_reads	Genotype	Allele read Ratio	A	C	G	T	Evidence for copy number loss
rs2736627	Ctrl DNA	Chr10	89721094	C	T	4312	299676	T/T	0.9858	41	4312	173	299676	
rs2736627	P2_CR	Chr10	89721094	C	T	154448	145284	C/T	**0.4847**	51	154448	132	145284	
rs2736627	P2	Chr10	89721094	C	T	123129	530187	C/T	**0.8115**	80	123129	362	530187	**YES***
rs2736627	P6	Chr10	89721094	C	T	7766	397232	T/T	0.9808	58	7766	253	397232	non-informative
rs701848	Ctrl DNA	Chr10	89726745	T	C	347811	1504	T/T	0.0043	129	1504	365	347811	
rs701848	P2_CR	Chr10	89726745	T	C	198875	96362	C/T	**0.3264**	161	96362	283	198875	
rs701848	P2	Chr10	89726745	T	C	155733	293234	C/T	**0.6531**	364	293234	254	155733	**YES***
rs701848	P6	Chr10	89726745	T	C	35164	276283	C/T	0.8871	289	276283	156	35164	**YES****
rs1234213	Ctrl DNA	Chr10	89689321	G	A	74251	69217	G/A	0.4825	69217	50	74251	28	
rs1234213	P2_CR	Chr10	89689321	G	A	196442	160	G/G	0.0008	160	6	196442	33	
rs1234213	P2	Chr10	89689321	G	A	39	2	failed	0.0488	2	0	39	0	non-informative
rs1234213	P6	Chr10	89689321	G	A	162432	1178	G/G	0.0072	1178	8	162432	17	non-informative
rs1234224	Ctrl DNA	Chr10	89675296	A	G	187029	170892	A/G	0.4775	187029	203	170892	323	
rs1234224	P2_CR	Chr10	89675296	A	G	412405	1089	A/A	0.0026	412405	338	1089	441	
rs1234224	P2	Chr10	89675296	A	G	889901	2079	A/A	0.0023	889901	680	2079	930	non-informative
rs1234224	P6	Chr10	89675296	A	G	608682	3702	A/A	0.0060	608682	527	3702	643	non-informative
rs74146236	Ctrl DNA	Chr10	89705221	A	G	399324	992	A/A	0.0025	399324	210	992	56	
rs74146236	P2_CR	Chr10	89705221	A	G	389274	764	A/A	0.0020	389274	223	764	39	
rs74146236	P2	Chr10	89705221	A	G	732426	1740	A/A	0.0024	732426	484	1740	110	non-informative
rs74146236	P6	Chr10	89705221	A	G	573884	1386	A/A	0.0024	573884	354	1386	99	non-informative
rs3895069	Ctrl DNA	Chr10	89725454	T	G	273784	99	T/T	0.0004	29	48	99	273784	
rs3895069	P2_CR	Chr10	89725454	T	G	307710	63	T/T	0.0002	12	36	63	307710	
rs3895069	P2	Chr10	89725454	T	G	519848	103	T/T	0.0002	14	46	103	519848	non-informative
rs3895069	P6	Chr10	89725454	T	G	374651	103	T/T	0.0003	24	43	103	374651	non-informative

Supplementary Table S4: Analysis of allelic read counts at known SNP positions within the PTEN locus supports the presence of deletions in samples P2 and P6

* Allelic read ratios from sample P2 differ significantly from those of its matched normal samples P2_CR, confirming the presence of a subclonal deletion of the "C" allele at this locus

** Sample P6 was non-informative (homozygous) for 5 of 6 SNPs. However, SNP rs701848 shows a significant fraction of "T" reads, suggesting that P6 is heterozygous (C/T) for this SNP and exhibits subclonal copy number loss at this locus

Allelic reads representing <0.5% of total read counts and likely to represent PCR error and were not considered "real"
